# The Alpha-defensin Test for Periprosthetic Joint Infections Is Not Affected by Prior Antibiotic Administration

**DOI:** 10.1007/s11999-016-4726-2

**Published:** 2016-02-10

**Authors:** Alisina Shahi, Javad Parvizi, Gregory S. Kazarian, Carlos Higuera, Salvatore Frangiamore, Joshua Bingham, Christopher Beauchamp, Craig Della Valle, Carl Deirmengian

**Affiliations:** The Rothman Institute at Thomas Jefferson University Hospital, Philadelphia, PA USA; Department of Orthopaedic Surgery, Rush University Medical Center, Chicago, IL USA; Department of Orthopaedic Surgery, Cleveland Clinic, Cleveland, OH USA; Department of Orthopaedic Surgery, Mayo Clinic Phoenix, Phoenix, AZ USA; The Lankenau Institute for Medical Research, 100 Lancaster Avenue, Wynnewood, PA 19096 USA

## Abstract

**Background:**

Previous studies have demonstrated that the administration of antibiotics to patients before performing diagnostic testing for periprosthetic joint infection (PJI) can interfere with the accuracy of test results. Although a single-institution study has suggested that alpha-defensin maintains its concentration and sensitivity even after antibiotic treatment, this has not yet been demonstrated in a larger multiinstitutional study.

**Questions/purposes:**

(1) For the evaluation of PJI, is prior antibiotic administration associated with decreased alpha-defensin levels? (2) When prior antibiotics are given, is alpha-defensin a better screening test for PJI than the traditional tests (erythrocyte sedimentation rate [ESR], C-reactive protein [CRP], fluid white blood cells, fluid polymorphonuclear cells [PMNs], and fluid culture)?

**Methods:**

This retrospective study included data from 106 hip and knee arthroplasties with Musculoskeletal Infection Society-defined PJI from four centers. Of the 106 patients in this study, 30 (28%) were treated with antibiotics for PJI before diagnostic workup (ABX group), and 76 (72%) were not treated before the diagnostic workup (NO-ABX group). There were no differences in age, sex, joint, culture-negative rate, or bacteriology between groups. The patients in the ABX group had antibiotics initiated by physicians who commenced care before assessment for PJI by the treating surgeon’s service. We compared the alpha-defensin levels and sensitivity between the ABX and NO-ABX groups. Additionally, the sensitivity of the alpha-defensin test was compared to that of traditional tests for PJI among patients on antibiotics.

**Results:**

The administration of antibiotics before performing the alpha-defensin test for PJI was not associated with a decreased median alpha-defensin level (ABX group, median 4.2 [range, 1.79–12.8 S/CO] versus NO-ABX, median 4.9 [range, 0.5–16.8 S/CO], difference of medians: 0.68 S/CO [95% confidence interval {CI}, −0.98 to 1.26], p = 0.451). Furthermore, the alpha-defensin test had a higher sensitivity (100%; 95% CI, 88.4%–100.0%) in diagnosing PJI among patients on antibiotics when compared with the ESR (69.0% [95% CI, 49.17%–84.72%], p = 0.001), the CRP (79.3% [95% CI, 60.3%–92.0%], p = 0.009), the fluid PMN% (79.3% [95% CI, 60.3%–92.0%), p = 0.009), and fluid culture (70.0% [95% CI, 50.6%–85.3%], p = 0.001).

**Conclusions:**

The alpha-defensin test maintains its concentration and sensitivity for PJI even in the setting of antibiotic administration. Furthermore, among patients with PJI on antibiotics, the alpha-defensin tests demonstrated a higher sensitivity in detecting PJI when compared with the ESR, CRP, fluid PMN%, and fluid culture. The high sensitivity of the alpha-defensin test, even in the setting of prior antibiotic treatment, provides excellent utility as a screening test for PJI.

**Level of Evidence:**

Level III, diagnostic study.

## Introduction

In the absence of major criteria such as a communicating sinus tract or two positive cultures, clinicians must rely on laboratory values to diagnose periprosthetic joint infection (PJI) [12]. We have previously demonstrated that premature antibiotic administration can compromise the sensitivity of traditional diagnostic laboratory results [13]. To increase the sensitivity of traditional diagnostic tests, the clinical practice guideline of the American Academy of Orthopaedic Surgeons recommends withholding antimicrobials for at least 2 weeks before aspiration of the joint [7]. Nevertheless, patients with possible PJI are often administered antibiotics before the treating surgeon has been consulted to initiate a diagnostic workup.

The alpha-defensin test has shown promising results for diagnosing PJI, as several independent institutions have demonstrated that the overall sensitivity and specificity of the alpha-defensin test is greater than 95% [2, 5, 8]. While the accuracy of the serum erythrocyte sedimentation rate (ESR), serum C-reactive protein, synovial fluid white blood cell (WBC) count, and polymorphonuclear (PMN) percentage tests are diminished in the setting of prior antibiotic administration [13], the sensitivity of the biomarker-based alpha-defensin test does not appear to be impacted [5]. However a larger multi-institutional study has not yet demonstrated the comparative alpha-defensin levels and sensitivity among patients treated with or without antibiotics before diagnostic testing.

We therefore asked: (1) For the evaluation of PJI, is prior antibiotic administration associated with decreased alpha-defensin levels? (2) When prior antibiotics are given, is alpha-defensin a better screening test for PJI than the traditional tests (erythrocyte sedimentation rate [ESR], C-reactive protein [CRP], fluid white blood cells [WBCs], fluid polymorphonuclear cells [PMNs], and fluid culture)?

## Materials and Methods

This retrospective diagnostic study was approved by the institutional review board. Four institutions collected synovial fluid, between October 2009 and July 2014, to study the diagnostic profile of the alpha-defensin test. Of 498 clinically annotated synovial fluid samples retrospectively identified as having an alpha-defensin test completed, 113 samples met the criteria for PJI and 385 samples did not meet the criteria for PJI. Inclusion required (1) the presence of a total joint arthroplasty; (2) sufficient data to categorize by the Musculoskeletal Infection Society (MSIS) criteria for PJI (Table 1); and (3) testing for alpha-defensin. Although some patients were missing individual laboratory tests used in the MSIS criteria for PJI, all patients in the study had sufficient laboratory results to meet the MSIS criteria for PJI. Patients with early postoperative PJI (4 weeks) were excluded, because synovial and serologic markers are not reliable parameters for the diagnosis of PJI in this setting [1, 11].

**Table 1:** Definition of PJI according to the ICM workgroup and the threshold for the minor diagnostic criteria

PJI is present when one of the major criteria or three out of five minor criteria exist:		
Major criteria	(1) Two positive periprosthetic cultures with phenotypically identical microorganism OR (2) A sinus tract communicating with the joint OR	
Minor criteria		Chronic PJI (>90 days)
	(1) Elevated serum CRP AND ESR	10 mg/L 30 mm/hr
	(2) Elevated SF WBC count OR Changes in the leukocyte esterase strip	3000 cells/μL + Or ++
	(3) Elevated SF PMN%	80%
	(4) Positive histological analysis of the periprosthetic tissue	> 5 neutrophil per high-power field in 5 high-power fields (×400)
	(5) A single positive culture	

PJI = periprosthetic joint infection; ICM = International Consensus Meeting; CRP = C-reactive protein; ESR = erythrocyte sedimentation rate; SF = synovial fluid; WBC = white blood cell; PMN = polymorphonuclear cells.

For the purposes of this specific study, we queried the clinical and electronic records of the 113 patients with PJI to determine whether intravenous and/or oral antibiotics were administered within 2 weeks before the diagnostic workup (joint aspiration and serologic marker measurements) for PJI. The records for seven patients were incomplete, leaving 106 patients included in this study. The mean patient age was 65 years including 41 women and 65 men. The synovial fluid samples were aspirated from 77 TKAs and 29 THAs.

Based on the antibiotic administration status, patients were allocated into two groups. The ABX (antibiotics) group includes patients who received antibiotics within 2 weeks before the diagnostic workup and the NO-ABX group includes patients who did not receive antibiotics before the diagnostic workup. Of the 106 patients with PJI included in this study, 30 (28%) patients comprised the ABX group and 76 (72%) patients comprised the NO-ABX group (Table 2). The patients in the ABX group were placed on antibiotics by a variety of emergency room physicians, primary medical physicians, and orthopaedic surgeons before being evaluated and tested by the treating surgeon’s service. Therefore, we were not able to ascertain the reasons for prediagnostic commencement of antibiotic administration. We were also unable to specify the particular antibiotic, dosage, or timing for these patients given that these details were spread among many hospitals near those included in this study. It is the general policy of all four institutions participating in this study to initiate a diagnostic workup before antibiotic treatment begins.

**Table 2:** Demographics of the patients included in our study

Patients	ABX (N = 30)	NO-ABX (N = 76)	p value
Age (years)	64.5 (range, 35–91)	65.8 (range, 25–89)	0.553
Gender	10 women, 20 men	31 women, 45 men	0.514
Joint (knee/hip)	21 knees/9 hips	56 knees/20 hips	0.809
Gram (+) organism (%)	71% (15/21)	76% (39/51)	0.156
Culture-negative PJI	30% (9/30)	33% (25/76)	0.172

ABX = antibiotics; PJI = periprosthetic joint infection

As expected, the majority of the isolated organisms in this study were Gram-positive bacteria including *Staphylococcus aureus* and *Staphylococcus epidermidis.* Overall 32% (34 of 106) of the patients in our cohort had culture-negative PJI, as defined by the MSIS criteria.

The demographic data between the ABX and NO-ABX groups did not demonstrate any significant differences. Specifically, there was no difference in age, sex, gender, culture-negative rate, or bacteriology between groups (Table 2).

The synovial fluid alpha-defensin level, ESR, CRP, fluid WBCs, and fluid PMN% were recorded for each patient. The median for each test was calculated for both groups. Additionally, the sensitivities of the tests were calculated using the MSIS criteria cutoff values as a standard to define the presence or absence of PJI (Table 1) [12].

### Statistical Analysis

Descriptive statistics were used to report all laboratory values. The one-tailed Mann-Whitney test (Prism; GraphPad Software, La Jolla, CA, USA) was used to determine whether the median laboratory value of any given test was significantly lower in the setting of prior antibiotic treatment as well as the 95% confidence interval between the difference. The two-tailed Fisher’s exact test was used to determine how the sensitivity of the alpha-defensin test compared with the sensitivity of ESR, CRP, fluid WBC, fluid PMN%, and culture in the setting of antibiotics. A p value of < 0.05 was considered statistically significant. A post hoc power analysis including the number of patients in this study was performed. The study has 80% power, at an alpha of 0.05, to identify a difference of 1.92 S/CO alpha-defensin level.

## Results

**Fig. 1:**
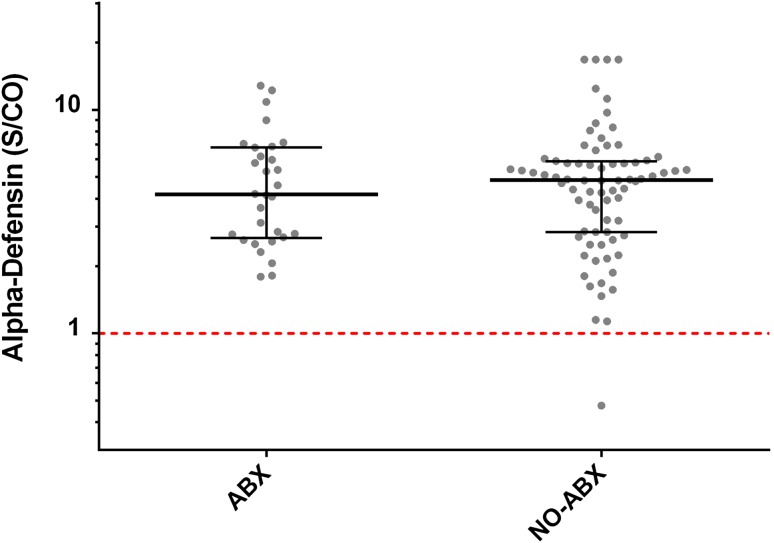
The alpha-defensin levels are graphed on a logarithmic scale for patients in the ABX and NO-ABX groups. The red line marks the positive threshold for the alpha-defensin test (signal/cutoff = 1). The black lines denote median group values with interquartile ranges. ABX = antibiotics group; NO-ABX = no antibiotics group; S/CO = signal-to-cutoff ratio.

The administration of antibiotics before performing the alpha-defensin test for PJI was not associated with a decrease in the median alpha-defensin level (ABX group, median 4.2 [range, 1.8–12.8 S/CO] versus NO-ABX, median 4.9 [range, 0.5–16.8 S/CO], difference of medians 0.68 S/CO [95% confidence interval {CI}, −0.98 to 1.26], p = 0.451, Fig. 1). Likewise, there was no associated decrease in the median ESR (ABX group, median 62 [range, 3–140 mm/hr] versus NO-ABX, median 65 [range, 1–140 mm/hr]; difference of medians 3 mm/hr [95% CI, −11 to 22 mm/hr, p = 0.252). However, the administration of antibiotics before diagnostic testing was associated with a decrease of the median CRP (25.7 mg/L [range, 1–302] in the ABX group versus 62.0 mg/L [range, 3–535) in the NO-ABX group; difference of medians 36.3, p = 0.008), the fluid WBCs (17,325 cells/μL [range, 413–104,200] in the ABX group versus 29,404 cells/μL [range, 1100–356,000] in the NO-ABX group; difference of medians = 12,079, p = 0.008), and PMN% (87% [range, 3–100] in the ABX group versus 92% [range, 40–100] in the NO-ABX group; difference of medians 5, p = 0.034) (Table 3).

**Table 3:** The median levels for alpha-defensin, ESR, CRP, fluid WBCs, and fluid PMNs in the ABX and NO-ABX groups

Diagnostic	ABX group (n = 30) Median (range)	NO-ABX group (n = 76) Median (range)	Difference (95% CI)	p value
Alpha-defensin (S/CO)	4.2 (1.8–12.8)	4.9 (0.5–16.8)	0.68 (−0.98 to 1.26)	0.451
ESR (mm/hr)	62 (3–140)	65 (1–140)	3 (−11 to 22)	0.252
CRP (mg/L)	25.7 (1.0–302)	62.0 (3.0–535)	36.3 (4.0–56.2)	0.008*
WBC (cells/μL)	17,325 (413–104,200)	29,404 (1100–356,000)	12,079 (1915–22,650)	0.008*
PMN (%)	87 (3–100)	92 (40–100)	5.0 (0.0–7.0)	0.034*

*Statistical significance; ESR = erythrocyte sedimentation rate; CRP = C-reactive protein; WBCs = white blood cells; PMN = polymorphonuclear; ABX = antibiotics; CI = confidence interval.

**Fig. 2:**
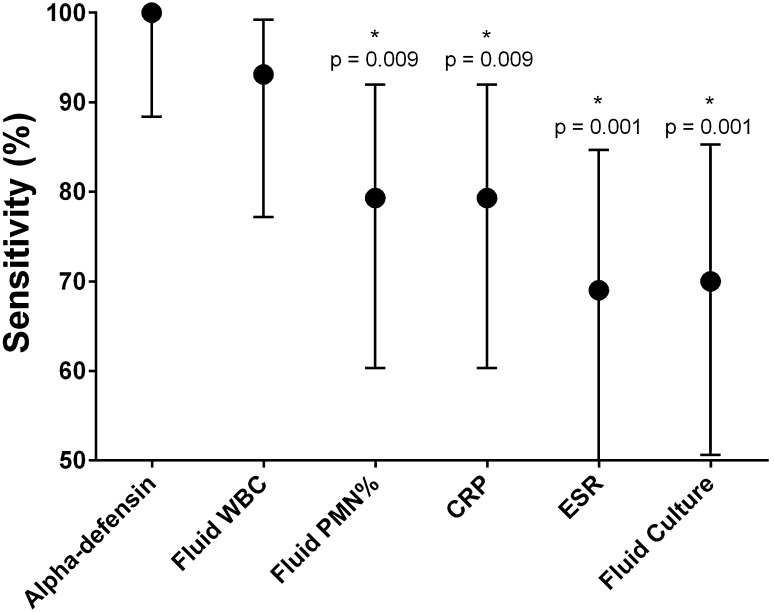
Comparison of diagnostic sensitivities of laboratory tests among patients treated with antibiotics before diagnostic testing for PJI. The asterisks denote tests that demonstrated a statistically significant lower sensitivity when compared with the alpha-defensin sensitivity.

The alpha-defensin test had improved sensitivity (100%; 95% CI, 88.4%–100.0%) among patients who were treated with antibiotics before diagnostic testing for PJI when compared with the ESR (69.0% [95% CI, 49.17%–84.72%], p = 0.001), the CRP (79.3% [95% CI, 60.3%–92.0%], p = 0.009), the fluid PMN% (79.3% [95% CI, 60.3%–92.0%], p = 0.009), and fluid culture (70.0% [95% CI, 50.6%–85.3%], p = 0.001) (Fig. 2). There was no difference when compared with the sensitivity of the fluid WBCs (93.1% [95% CI, 77.2%–99.2%], p = 0.147).

## Discussion

Our previous study showed that the traditional diagnostic tests for PJI can be compromised by premature antibiotic administration [13]. Although numerous studies have demonstrated that the synovial fluid alpha-defensin test is likely the most accurate single test for PJI [3, 4, 6, 9], only one single-institution study has suggested that antibiotic treatment is not associated with a decrease in alpha-defensin sensitivity. The goal of our multiinstitution study was to ascertain whether antibiotic treatment before diagnostic testing would be associated with decreased alpha-defensin sensitivity and to compare this sensitivity with other traditional tests for PJI.

There are several limitations to our study and our findings should be interpreted in light of these shortcomings. Many patients were referred to the tertiary care centers in this study and did not have detailed accompanying information regarding the start date or rationale behind the initiation of antibiotics. It would have been ideal to evaluate the rationale for antibiotic initiation and the duration of treatment in relation to the results in this study. Although we were able to demonstrate no difference between certain demographic characteristics in the two groups, there is always a possibility that an unknown factor, other than antibiotic treatment, caused some of the diagnostic differences identified in this study. Another limitation relates to the use of the MSIS criteria for PJI. It is possible that some patients with PJI do not meet the MSIS criteria, which would have resulted in a failure to include them in this study. We believe that this population of patients with a false-negative MSIS result is likely very small and would not have perturbed our current results. Additionally, we only assessed patients with PJI to assess the sensitivity of alpha-defensin and did not intend to assess specificity with and without antibiotics. Finally, patients were identified from four institutions based on having had an alpha-defensin test request, and seven of 113 identified patients were excluded as a result of incomplete medical records. It is certainly possible, as a result of the circumstances of admitting patients with PJI to the hospital, that some PJIs cared for by this study’s investigators were not recruited for this study. Although we cannot assess how many patients were not included, we have no reason to believe that there was any selection bias in the population of patients included in this study.

Our multiinstitution study demonstrates that the initiation of antibiotic treatment for PJI before diagnostic evaluation does not appear to be associated with decreases in the median alpha-defensin levels. In fact, the consistency of the median alpha-defensin level in this study was observed among a population of patients on antibiotics who simultaneously demonstrated a decreased median CRP, fluid WBCs, and fluid PMNs. These results corroborate the single-institution findings of Deirmengian et al. [5], who did not observe any effect of prior antibiotic treatment on alpha-defensin levels or sensitivity. They also corroborate the results of a previous study demonstrating the fact that many traditional tests for PJI have lower mean levels among patients with PJI who started antibiotic treatment before diagnostic testing [13]. Although it is possible that a higher powered study may demonstrate a statistically significant difference in alpha-defensin levels among those on antibiotics, the small absolute difference identified in this study would require a study of over 8000 PJIs to demonstrate that this difference is statistically significant.

This study also found that when screening for PJI in the setting of antibiotic use, alpha-defensin is more sensitive than the ESR, CRP, fluid PMN%, and fluid culture. Given the numbers in this study, we did not find a statistically significant improvement over the fluid WBCs. The importance of any screening test lies in its ability to identify disease reliably, thus having a high sensitivity. Several previous studies have demonstrated the sensitivity of the alpha-defensin test to be greater than 95%. Deirmengian et al. [6] reported that the alpha-defensin levels were consistent results regardless of the organism type, Gram type, species, or virulence of the organism. In another study by Bingham et al. [2], the authors concluded that the sensitivity and specificity of the synovial fluid alpha-defensin assay exceeded the sensitivity and specificity of other currently available clinical tests. In this study we extend the utility of the alpha-defensin test to screening those patients who were started on antibiotic treatment before diagnostic testing. The appropriate use of the alpha-defensin test remains to be definitively established. One might suggest that the alpha-defensin test should only be used when traditional tests fail to provide a clear diagnosis, promoting cost-effectiveness. On the other hand, the alpha-defensin test has been consistently demonstrated by several institutions to be the most sensitive and specific individual test for PJI [2, 9, 10] and also has a negligible cost relative to the care of PJI (approximately USD 31 reimbursement by the Centers for Medicare & Medicaid Services for the laboratory-based Synovasure PJI test; CD Diagnostics, Claymont, DE, USA). Selective utilization of the alpha-defensin test only after receiving traditional test results would require reaspiration of the joint and presupposes the ability to differentiate between clear and ambiguous cases of PJI, which is likely not consistent among surgeons. The appropriate use of the alpha-defensin test in practice is currently being defined by individual institutions.

In summary, our study demonstrates that alpha-defensin maintains its synovial fluid levels even when patients are treated with antibiotics before a diagnostic workup. Additionally, among patients treated with antibiotics before diagnostic testing, the alpha-defensin test had a higher sensitivity and provided better screening for PJI than the ESR, CRP, fluid PMN%, and fluid culture.
